# Supplementary material for: Characterizing Early Cardiac Metabolic Programming via 30% Maternal Nutrient Reduction during Fetal Development in a Non-Human Primate Model
Source: Int J Mol Sci. 2023 Oct 14;24(20):15192. doi: 10.3390/ijms242015192 (PMC10607248; doi:10.3390/ijms242015192)
Supplement: Supplementary file 1 [file ijms-24-15192-s001.zip › ijms-2576000-supplementary.pdf]

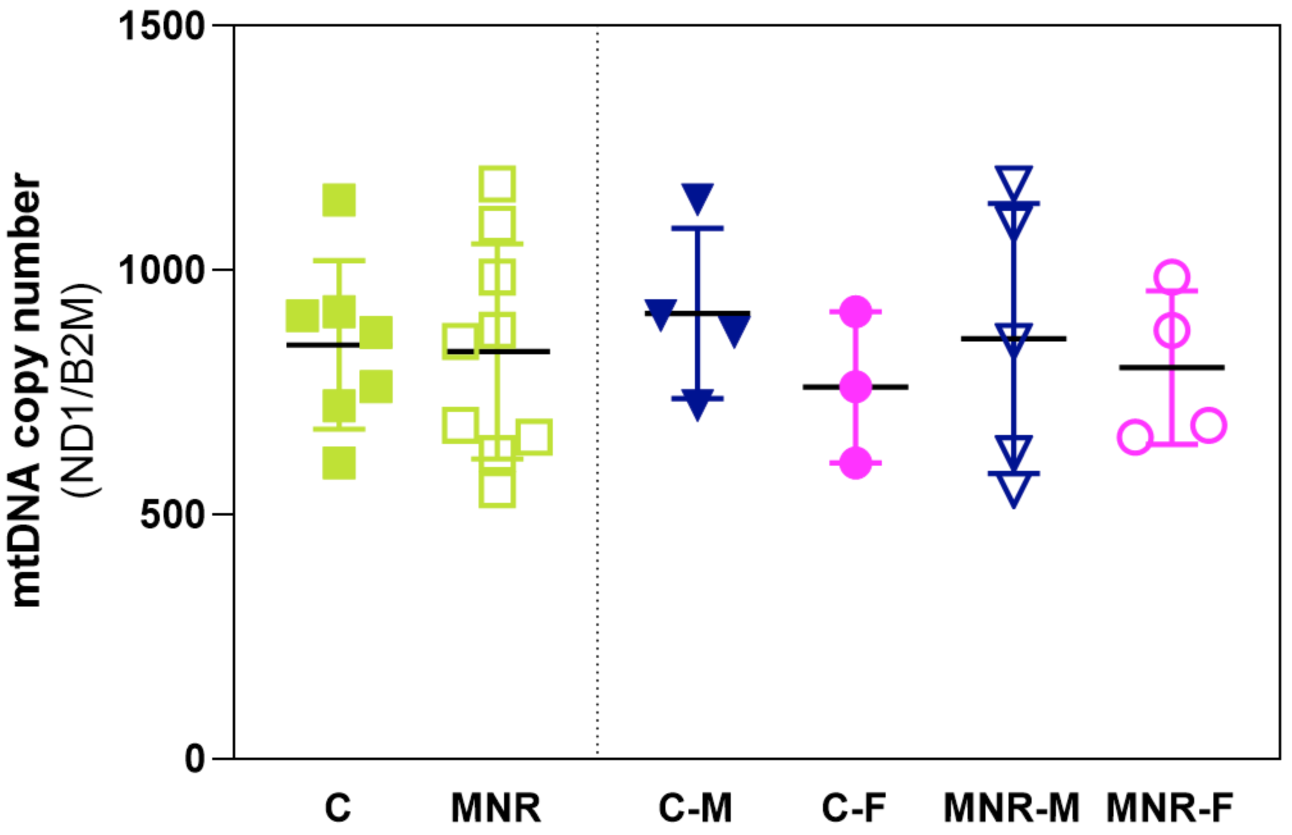

Figure S1. Cardiac relative mtDNA copy numbers of mitochondrial genes.
Control (C): fetuses born from mothers fed a control diet; MNR: fetuses born from mothers fed a 30% nutrient-restricted diet; C-M/C-F: male/female fetuses born from mothers fed a control diet (filled symbols); MNR-M/MNR-F: fetuses born from mothers fed a 30% nutrient-restricted diet (open symbols).
